# Empathy and Interest Towards an Autistic Person and the Effect of Disclosing the Diagnosis

**DOI:** 10.1007/s10803-025-06802-2

**Published:** 2025-04-09

**Authors:** Yonat Rum, Shir Genzer, Ofer Golan, Carrie Allison, Simon Baron-Cohen, Anat Perry

**Affiliations:** 1 Seymour Fox School of Education, The Hebrew University of Jerusalem, Jerusalem, Israel; 2 Department of Psychology, The Hebrew University of Jerusalem, Jerusalem, Israel; 3 Department of Psychology, Bar-Ilan University, Ramat Gan, Israel; 4 Autism Research Centre, Department of Psychiatry, University of Cambridge, Cambridge, UK

**Keywords:** Autism, Self-disclosure, Empathy, Empathic accuracy, Double empathy

## Abstract

This study investigates the effects of disclosing an autism diagnosis on non-autistic listeners’ empathy and social interest towards the autistic discloser. In Study 1, participants (non-autistic students in the social sciences/humanities [*n* = 99; 70% female]) watched a video of an autistic adult sharing an autobiographical story and reported how they believed the storyteller felt, following an introduction in which the storyteller did or did not disclose their diagnosis. Their evaluation of the storyteller’s emotions was compared to the storyteller’s own reports, resulting in an empathic accuracy measure. Participants reported how empathic they felt towards the storyteller and how socially interested they were in them. Studies 2 and 3 replicated the same procedure with STEM students (*n* = 96; 40% female), and with non-student adults (*n* = 76; 50% female) from diverse professional/occupational backgrounds, with an additional question about working together. In Study 1, participants in the self-disclosure condition demonstrated higher empathic accuracy, reported more empathy, and greater social interest in the storyteller. Study 2 showed a similar trend of higher empathy in the self-disclosure condition but no differences in social interest measures. Interest in working with the storyteller was higher in the self-disclosure condition. In Study 3, participants in the self-disclosure condition demonstrated higher empathy and greater interest in hearing another story and working with the storyteller. An individual’s self-disclosure of an autism diagnosis improved others’ ability to empathize with them and willingness to work with them. We discuss the complex effect of self-disclosure on social interest in an autistic person.

The theory that autistic people struggle with empathy, that is, understanding others’ mental states and responding to these with a similar or appropriate emotion, has offered an explanation for the social communication challenges in autism (Baron-Cohen, 2009; Baron-Cohen & Wheelwright, 2004; Greenberg et al., 2018). However, a new perspective, the ‘double empathy problem’ theory (Milton, 2012; Milton et al., 2022), reframed these social communication difficulties as a breakdown in mutual understanding between autistic and non-autistic people. According to this theory, first proposed by Milton (2012), the ‘empathy problem’ in autism was historically framed as an individual issue when it should be considered as an interpersonal issue.

To understand this breakdown, communication flows optimally when each person in an interaction is tracking the mental states (intentions, beliefs, desires, and emotions) of the interlocutor (Grice, 1989). Specifically, earlier works showed that autistic people struggle with adhering to the implicit “Griceean Maxims” in conversion, such as “Be Relevant”, “Be Truthful”, “Be Informative”, and “Be Clear” (Surian et al., 1996). This communication pattern is one way in which difficulties in ‘Theory of Mind’ (ToM) can be manifested in expressive speech to cause difficulties in the pragmatics of language (Barnes & Baron-Cohen, 2012; Baron-Cohen, 1988, 1997).

Milton’s key insight was to point out that when there are breakdowns in communication, the cause could be misunderstandings by both parties, not just one. Some theories might suggest that whilst the autistic person struggles with ToM for neurological reasons (Povinelli & Povinelli, 1996), a non-autistic listener might struggle with understanding the autistic speaker because their speech is confusing, perhaps exacerbated if the non-autistic listener does not know that the speaker is autistic. Others, derived from the double empathy problem framework, propose that both autistic and non-autistic people have equally valid experiences that are different and that the neurological differences accumulate in misunderstandings while neither party is inherently “correct”. Supporting this bidirectional view, recent research has focused on breakdowns in such mixed-neurotype social communication, indicating that autistic people better connect with other autistic people than they do with non-autistic people (Chen et al., 2021; Crompton et al., 2020; DeBrabander et al., 2019; Morrison et al., 2020; Schilbach, 2016).

An important implication of the double empathy problem theory is the importance of studying not only the empathic abilities of autistic individuals but also the empathy of non-autistic individuals *towards* them. The present study uses the double empathy problem as a theoretical framework to investigate how disclosing an autism diagnosis might influence non-autistic people’s empathy towards autistic individuals. To date, no studies have examined the effects of self-disclosure on empathy towards an autistic person.

The multifaceted concept of empathy includes cognitive empathy, defined as recognizing or inferring another’s mental state (ToM), and affective empathy, defined as responding to another’s mental state with an appropriate emotion (Baron-Cohen, 2011; Decety & Holvoet, 2021). Empathy is important for explaining and predicting other people’s behaviour and navigating the social world (Davis, 2004; Decety et al., 2016; Decety & Holvoet, 2021; Knafo-Noam et al., 2015). One effective way to measure empathy is through empathic accuracy paradigms, where researchers assess how well observers can track others’ emotional experiences. Zaki and Ochsner (2009) developed a particularly valuable approach that measures empathic accuracy by comparing perceivers’ interpretations of targets’ emotional states with the targets’ own reports of their feelings. This paradigm, further developed by Jospe et al. (2020), provides a more ecological measure of empathy by using real autobiographical emotional stories as stimuli, allowing researchers to capture both the cognitive and affective components of empathy in a naturalistic context.

Literature has begun to emerge on the difficulty of non-autistic people to empathize with autistic people. For example, in a paper focusing on mental state attribution based on perceiving animated movement, Edey et al. (2016) stated: “Typical adults exhibit mind-blindness towards those with autism.” (Edey et al., 2016). They used an adaptation of a task designed to interpret mental states based on movement, which is presented by animation (Abell et al., 2000; Heider & Simmel, 1944). Previous studies indicated that non-autistic individuals score higher than autistic individuals on this task (Abell et al., 2000; Castelli et al., 2002). Edey and colleagues created a version of this task with animations based on movement produced by typical people and people with a diagnosis of autism. They then compared the performance of autistic and typical participants, finding that typical participants were better at attributing mental states to the animations generated by the movements of other typical individuals relative to those generated by the movements of autistic individuals (note that when citing previous research, we maintain authors’ original terminology [e.g., “typical”, “non-autistic”] to preserve the specific context of their findings, while in presenting our own research and results in this paper, we will use the term “non-autistic”). Other studies also suggest that it is harder for typical participants to interpret the mental state of autistic individuals than of other typical individuals based on pictures of facial expressions (Brewer et al., 2016) or video-recorded naturalistic behavior (Sheppard et al., 2016).

In a recent study, Cheang et al. (Cheang et al., 2024) found that participants from the general population demonstrated significantly lower ‘empathic accuracy,’ i.e., the ability to accurately judge the mental states of others, when viewing autobiographical accounts of emotional events from autistic narrators, compared to non-autistic narrators. The researchers used video clips of autistic and non-autistic people recounting emotional events to test if participants from the general population could accurately track the narrators’ emotions. As predicted by the double empathy problem theory, they found it was harder for non-autistic participants to track autistic narrators’ emotions compared to tracking non-autistic narrators’ emotions. The participants in the study were unaware of the diagnostic status of the storytellers, and it could be that knowing that the person is autistic impacts the participants’ ability to accurately empathize with them. This question is examined in the present study.

Alongside research on the difficulties of non-autistic people in understanding autistic people, some findings also point to less social interest of typical people to interact with autistic people than with other typical people (Sasson et al., 2017). Recent studies suggest that in brief encounters or based on short videos, autistic individuals are perceived by typical people as less ‘likable,’ less credible, or more awkward than non-autistic individuals (Alkhaldi et al., 2021; Lim et al., 2022; Sasson et al., 2017). Thus, there is growing empirical support for the ‘empathy problem’ also being influenced by attributions made by the non-autistic person about the autistic person. However, there is a paucity of research focusing on what might impact the empathy and social interest of non-autistic people towards autistic people. Learning what could help non-autistic people better empathize with autistic people and have more interest in connecting with them may help to support mixed-neurotype social communication and may promote inclusion. In the present study, we investigated whether non-autistic participants’ empathy and social interest toward an autistic individual are impacted by the autistic person self-disclosing their diagnosis.

Self-disclosure generally promotes interpersonal connection, with research showing increased liking towards those who share personal information (Collins & Miller, 1994). However, in the context of autism, disclosure decisions involve complex considerations. Recent research has shown that autistic identity itself influences disclosure patterns, with those who feel their autistic identity is more central to their self-concept reporting more frequent disclosure (Love et al., 2023). Research with autistic college students has revealed that disclosure decisions are carefully weighed against whether they will support or inhibit understanding by others (Frost et al., 2019). Recognizing these complexities, recent support programs for autistic adults have focused on providing guidance around disclosure decisions (Han et al., 2024). Indeed, autistic people report that while disclosure can lead to greater acceptance and understanding from others, it also carries risks of stigma and discrimination (Lindsay et al., 2014; Linton, 2014).

Examining the impact on disclosure recipients, studies indicate that the disclosure positively affects affective responses and likeability (Brosnan & Mills, 2016; Maras et al., 2019) and leads to more favorable first impressions of typical people about autistic individuals (Sasson & Morrison, 2019). In a study that used video-recorded mock employment interviews to investigate the impact of diagnostic disclosure of an autistic interviewee on the evaluations of their performance, it was found that when raters knew that the candidates were autistic, they perceived them more favorably, compared to when they watched the interviewees without knowing that they were autistic (Norris et al., 2024). Although there is empirical support for greater liking and more positive judgments towards autistic individuals when disclosing their diagnosis, these may not necessarily translate to enhanced empathy (which includes the cognitive and affective aspects as defined above) towards them. For example, a favourable judgment could also result from other factors, such as social desirability.

It could also be that the disclosure of autism diagnosis negatively impacts empathic accuracy, as relying on categorical information might prevent us from understanding what is being communicated in a specific situation due to expectations and biases (Heasman & Gillespie, 2018; Macrae & Bodenhausen, 2000). Knowing that a person is autistic might trigger specific expectations, assumptions, or biases in the non-autistic person, which could add barriers instead of bridging the gap in empathy in mixed-neurotype communication.

In summary, the existing literature supports the double empathy problem theory in terms of the difficulties of non-autistic people in empathizing with autistic people, but to the best of our knowledge, it has not yet been examined if knowing that a person is autistic impacts the ability of non-autistic people to accurately empathize with the autistic person. The literature also supports the hypothesis that the disclosure of autism diagnosis positively impacts liking towards the autistic discloser on the part of non-autistic people (Brosnan & Mills, 2016; Maras et al., 2019; Norris et al., 2024; Sasson & Morrison, 2019) but it has not yet been investigated whether self-disclosure directly impacts *empathy* towards the autistic person.

In the present study, we aimed to address these gaps. We investigated whether self-disclosing an autism diagnosis affects empathy and social interest towards an autistic person on the part of non-autistic individuals, and, if so, in what direction.

To investigate these questions, we created a stimulus based on an autistic person’s lived experience. We then conducted three studies in which data were collected from non-autistic participants who were asked to respond to the stimulus. In Study 1, we investigated non-autistic participants studying humanities/social sciences empathy and social interest towards an autistic person, and the role of disclosing an autism diagnosis in these processes. In Study 2, we investigated the same questions with participants who were students in STEM (Science, Technology, Engineering, Mathematics) to examine the generalisability of the initial findings and to learn about possible similarities and differences in the responses to autism self-disclosure on the part of non-autistic individuals with different characteristics. We also added an additional question regarding the willingness of participants to hypothetically work with the autistic individual. In Study 3, we aimed to replicate the results outside a University context with non-student adult participants from the general population. The procedure was approved by the Hebrew University Ethics Committee. Informed consent was obtained from all participants.

## Study 1

### Methods

#### Stimulus Development

We replicated a procedure devised by Zaki and Ochsner (Zaki & Ochsner, 2009) and Jospe and colleagues (Jospe et al., 2020) to measure empathic accuracy (EA) (Ickes, 1993). A measure of EA is generated by computing the concordance between a perceiver’s (the subject of the EA measure) view of a target (the object of the EA measure) and the target’s own report on their internal states. An EA paradigm is based on the perceiver’s interpretation of a target’s videotaped autobiographical story as the stimulus and the correspondence between the perceiver’s and the target’s ratings of how the target felt while storytelling. (Genzer et al., 2022; Jospe et al., 2020; Zaki & Ochsner, 2009, 2011). Previous studies that have used this paradigm, including those focusing on non-typical populations, relied mainly on typical storytellers as targets, i.e., target participants that did not have any neurodivergence, health or mental health conditions (for a review on the use of empathic accuracy paradigms in studies on non-typical populations see: Rum & Perry, 2020). In the present research, the target was an autistic person. Other adaptations to the procedure were made following consultation with an advisory committee of 5 autistic adults. These included phrasing instructions, reviewing language, and adding questions to collect qualitative data (not reported here).

To create the stimulus, an autistic adult (a 35-year-old male; henceforward, will be referred to as the “storyteller”) was video-recorded online using the *Zoom* platform, sharing autobiographical memories, resulting in three clips. After the recording, the storyteller was asked to watch each clip and continuously rate his feelings’ positive to negative valence using a computerized rating slider. At the end of each clip, he was asked to rate the degree to which he felt 12 emotions (embarrassment, anger, sadness, happiness, disgust, pride, fear, excitement, contentment, stress, calmness, and alertness) on a scale of 0 (“Not at All”) to 8 (“Very Much”). In the end, he was asked to specify whether he gave the research team permission to use each story in future research. All ratings and reports were collected using the *Qualtrics* platform. The story used as the stimulus in the present study described his experience of a past romantic relationship that had ended and the storyteller’s feelings that he might not have the opportunity to experience such a relationship again (the length of the video was 1 min 43 s).

To allow the creation of two experimental conditions into which future participants would be assigned (see methods), the storyteller recorded two short introductions: In one, he stated that the story he was about to share was an authentic lived experience (no-disclosure statement). In the second, he stated that the story is an authentic experience and added that he is autistic (self-disclosure statement).

#### Participants

Participants were recruited via the students’ recruitment platform at the Hebrew University and social media. Nine participants (from a total of 108) were excluded (5 reported technical problems, 3 reported not being native speakers of the language used, and one reported unusual noise during participation), resulting in a final sample of 99 participants (70.71% female, 29.29% males, *M*_age_=24.02, *SD*_age_=3.2), all students studying psychology, social work, education, and other social sciences and/or humanities subjects, all non-autistic, according to their own reporting.

#### Procedure and Design

Participants watched (via *Qualtrics*) an instructional video and completed a practice trial, followed by the EA task, in which they were asked to continuously rate how positive or negative they believed the storyteller felt while sharing the story as they were watching it and to rate the degree to which they think the storyteller felt each of the 12 emotions and answer six questions about their experience (see measures). Before viewing and rating the story, participants were randomly assigned to one of two conditions: (1) *Self-disclosure* condition (*n* = 50; 72% females), which included the pre-recorded self-disclosure statement, or (2) *No-disclosure* condition (*n* = 49; 67% females), which included the no-disclosure statement.

### Measures

#### Empathic Accuracy (EA)

##### Continuous Valence EA

This measure was calculated as the correlation between each participant and the storyteller’s continuous ratings of positive to negative feelings (of the storyteller) in each second of the video-recorded story (Genzer et al., 2022).

##### Specific Emotions EA

The distance (absolute value) between the participant’s rating and the storyteller’s rating for each of the 12 emotions was calculated, then this scale was reversed, so maximum accuracy in a given emotion received 8 points, and minimum accuracy received 0 points. The sum of all the reversed distances reflects the general emotion-recognition accuracy, from 0 to 96. This scale was then transformed into one between 0 and 100 for a more straightforward interpretation (Israelashvili et al., 2019). The aggregation of the 12 emotions into a single EA score follows established procedures in empathic accuracy research (Genzer et al., 2022; Israelashvili et al., 2019; Jospe et al., 2020). While each emotion was rated separately by both the storyteller and participants, combining them provides an overall measure of emotional understanding accuracy. This approach allows us to assess general empathic accuracy while maintaining statistical power and avoiding multiple comparison issues.

#### Self-reports

Participants were asked to rate each item on a scale of 0 (not at all) to 8 (to an extremely large extent):

To learn about their empathy, they were asked:

“To what extent did you feel empathy towards the storyteller?”

To learn about their social interest in the storyteller, they were asked:

“To what extent would you like to listen to another story from the storyteller?“.

“To what extent would you like to meet the storyteller if you had the opportunity?”

To learn about their listening, effort, and the attributed importance to their performance, they were asked the following questions, respectively:

“To what extent did you listen to the storyteller?“.

“To what extent did you make an effort to infer the storytellers’ emotions?“.

“To what extent would you be interested in knowing if you were accurate in inferring the storytellers’ emotions?”

### Data Analysis

Statistical analyses were performed using R software (R Core Team, 2020). Each analysis was conducted after removing outliers’ responses above or below 2.5 SD in the relevant dependent variable. Two-sample t-tests with Bonferroni correction for multiple comparisons were conducted to assess the difference in EA scores and the six self-reported items between the conditions. Separate adjustments were made for the behavioral Empathic Accuracy scores and the self-report measures.

### Results

The analyses revealed significant differences between the conditions for the specific emotions’ EA (*t*_(95.83)_ = 2.34, *p* = .021, *d* = 0.47), the self-reported empathy (*t*_(84.66)_ = 2.43, *p* = .017, *d* = 0.50), interest in listening to an additional story (*t*_(85.30)_ = 3.61, *p* = .001, *d* = 0.73), and in meeting the storyteller (*t*_(94.95)_ = 3.24, *p* = .002, *d* = 0.65), indicating higher specific emotions EA, self-reported empathy and social interest towards the autistic storyteller in the self-disclosure condition compared to the no-disclosure condition. The significance in the EA measure held after Bonferroni correction for multiple comparisons (*adjusted p* = .043), as well as the measures of social interest (listening to an additional story: *adjusted p* = .003; meeting the storyteller: *adjusted p* = .010), but the difference for self-reported empathy, did not (*adjusted p* = .104). Differences between the conditions in the continuous valence EA, self-reported listening, the effort to empathize, and attributed importance to performance were not statistically significant (Table 1 presents the full analysis).

**Table 1:** Comparisons between the Self-Disclosure and the No-Disclosure conditions for Empathic Accuracy and Self-Reports measures in study 1

	Parameter	Self-Disclosure (*n*)	No- Disclosure (*n*)	Mean Self-Disclosure	Mean No- Disclosure	df	t	*p*	Adjusted *p*	d
**Empathic Accuracy**	Continuous valence	49	47	0.71	0.66	88.39	1.12	0.267	0.534	0.23
	Specific emotions	50	48	60.00	57.77	95.83	2.34	**0.021**	**0.043**	0.47
**Self-Reports**	**Empathy**	50	45	6.94	6.31	84.66	2.43	**0.017**	0.104	0.50
	**Social interest**: Interest in listening to an additional story	50	49	5.90	4.45	85.30	3.61	**0.001**	**0.003**	0.73
	**Social interest**: Interest in meeting the storyteller	50	49	4.74	3.33	94.95	3.24	**0.002**	**0.010**	0.65
	**Listening**	48	48	7.40	7.25	93.99	1.02	0.312	1.000	0.21
	**Effort**	50	47	7.02	6.70	89.92	1.16	0.249	1.000	0.24
	**Attributed importance**: Interest in knowing how accurate you were	50	47	6.18	6.47	94.92	-0.84	0.401	1.000	0.17

* *Adjusted p*– p values after Bonferroni correction, with separate adjustments for the behavioral Empathic Accuracy scores and the self-report measures

### Discussion

These results align with the theoretical framework about the double empathy problem and the potential effects of disclosure on empathy towards an autistic person. Specifically, we found that an autistic person’s self-disclosure enhances empathy and social interest toward them. However, it did not affect the level of listening, the effort to empathize (though both show ceiling effects), or the attributed importance to one’s own performance in empathizing.

As discussed in our review of disclosure effects, the fact that participants in the self-disclosure condition scored higher in the self-reported empathy and social interest measures could be potentially explained by social desirability to appear rather than being more empathic towards the storyteller when knowing he is autistic. However, taken together with the improved accuracy in a behavioural measure (EA), the results indicate that autism self-disclosure positively impacts non-autistic people’s empathy towards an autistic person. Moreover, since one of the two questions measuring social interest was whether they would like to listen to an additional story by the storyteller, results imply that participants who heard the self-disclosure were willing to invest more time in the storyteller.

Importantly, participants were mainly students who studied subjects focused on human behaviour. Therefore, the increase in social interest in an autistic person and/or the increased social desirability could also be specific to this characteristic. We thus wanted to investigate whether the results replicate in a different population. Much research in social and behavioral sciences relies on samples similar to our Study 1 sample (this is because, in many universities, students in these fields are required to participate in experimental studies as part of their programs, making it easier for researchers to recruit them as participants). We, therefore, wanted to examine the generalisability of our initial findings in an additional sample of students. We designed Study 2 to investigate the same questions with non-autistic participants studying sciences, technology, engineering, or mathematics (STEM).

## Study 2

In this study, we aimed to investigate the same questions as in Study 1 in a different population of individuals who are not studying human behaviour. Study 2 also expands study 1 by adding an additional question related to a potential long-term impact of self-disclosure on the listener - the willingness to work with the autistic storyteller. Our main prediction was that results would be similar to those of Study 1, although they may be attenuated, as students in STEM have been previously shown to have lower empathy traits compared to students in the humanities and social sciences (Goldenfeld et al., 2005; Wakabayashi et al., 2006; Wheelwright et al., 2006). We had no a priori hypothesis regarding the willingness to work with the autistic individual.

### Methods

#### Participants

Participants were recruited through postings on social media groups of STEM students and email invitations distributed to students by the Hebrew University’s relevant departments. One hundred and five students completed the study procedure, and 9 of them were excluded from the sample (5 were not STEM students, one reported an autism diagnosis, one reported not being a native speaker of the language used, and 2 participants reported technical problems during participation). Final analyses were conducted on 96 non-autistic participants (39.58% females, 59.38% males, 1.04% other, *M*_age_=25.85, *SD*_age_=3.34). Participants were students studying computer science, engineering, physics, mathematics, chemistry, biology, statistics, biomedical sciences, economics, or sciences for high-tech.

#### Procedure and Design

The procedure and design were similar to that in Study 1, such that participants were randomly assigned to one of the two conditions: (1) *Self-Disclosure* (*n* = 52; 38% females) or (2) *No-Disclosure* (*n* = 44; 50% females).

#### Measures

Measures were identical to those used in Study 1, with the addition of one self-report question designed to measure social interest specifically in the context of a workplace (‘To what extent would you be interested in working with the storyteller at the same team in a workplace?‘).

#### Data Analysis

The data analysis procedure was identical to that used in Study 1.

### Results and Discussion

The results revealed a similar pattern to those of Study 1 in the measures of empathy, with marginal differences between the conditions for the specific emotions’ EA (*t*_(88.52)_ = 1.91, *p* =.060, *d* = 0.40) and the self-reported empathy (*t*_(89.05)_ = 1.82, *p* =.072, *d* = 0.38), and also for the continuous valence EA (*t*_(80.27)_ = 1.85, *p* =.068, *d* = 0.39), implying greater empathy towards the autistic storyteller in the self-disclosure condition compared to the no-disclosure condition. While the effects were consistently in the same direction, the effect sizes in this study were somewhat smaller, necessitating a larger sample size to achieve statistical significance. This suggests that the disclosure of autism diagnosis may indeed have an impact, albeit to a lesser degree, on individuals studying STEM compared to those in social sciences and humanities disciplines. The differences in effect sizes between Studies 1 and 2 might be partially explained by differences in knowledge about autism (for example, if social science students had more knowledge through their coursework, it potentially enabled them to better identify and understand autistic communication patterns when made aware of the diagnosis), or perhaps greater attention. Future studies should directly measure these potential moderating effects. The effect of self-disclosure on the measures of social interest was not replicated in Study 2, and there was no significant difference between the reports of participants in the self-disclosure condition and those in the no-disclosure condition. Each measure showed a positive trend, albeit not statistically significant. This implies that the self-disclosure statement could either improve social interest or have no significant impact, but importantly, we found no evidence that it reduced social interest.

The self-disclosure of the autism diagnosis led to more willingness to work with the autistic person: a significant difference was found between the two conditions for the added social interest measure focused on the workplace (*t*_(89.61)_ = 4.14, *p* <.001, *d* = 0.85 [*adjusted p* =.001]). Similar to study 1, differences between self-reported listening, the effort to understand the storyteller, and the interest in knowing how accurate you were (attributed importance to performance) were not statistically significant (see Table 2 for the full analysis).

**Table 2:** Comparisons between the Self-Disclosure and the No-Disclosure conditions for Empathic Accuracy and Self-Reports measures in study 2

	Parameter	Self-Disclosure (*n*)	No- Disclosure (*n*)	Mean Self-Disclosure	Mean No- Disclosure	df	t	*p*	Adjusted *p*	d
**Empathic Accuracy**	Continuous valence	50	42	0.71	0.63	80.27	1.85	*0.068*	0.135	0.39
	Specific emotions	51	43	61.38	58.43	88.52	1.91	*0.060*	0.112	0.40
**Self-Reports**	**Empathy**	50	42	6.42	5.81	89.05	1.82	*0.072*	0.507	0.38
	**Social interest**: Interest in listening to an additional story	52	44	4.65	4.09	92.00	1.14	0.256	1.000	0.23
	**Social interest**: Interest in meeting the storyteller	52	44	2.94	2.61	90.79	0.68	0.497	1.000	0.14
	**Social interest**: Interest in working with the storyteller in a workplace	52	44	5.02	3.48	89.61	4.14	**0.0001**	**0.001**	0.85
	**Listening**	52	42	7.27	7.17	85.45	0.49	0.625	1.000	0.10
	**Effort**	51	42	6.69	6.48	80.41	0.69	0.492	1.000	0.15
	**Attributed importance**: Interest in knowing how accurate you were	50	43	6.24	6.09	88.17	0.38	0.702	1.000	0.08

*Adjusted p– p values after Bonferroni correction, with separate adjustments for the behavioral Empathic Accuracy scores and self-report measures

These results imply an overall trend of a positive effect of self-disclosure on empathy towards an autistic person. However, while results were in the same general direction, they were not significant, suggesting that the impact of self-disclosure on empathy and social interest towards an autistic person may be moderated by individual differences among participants (studying STEM vs. humanities/social sciences).

Interestingly, participants in Study 2 demonstrated improved EA in the continuous measure (marginal significance and a larger effect size than in Study 1) in addition to the specific emotions measure. On the other hand, they did not demonstrate an enhanced self-reported interest in hearing another story or meeting the storyteller when knowing that he is autistic, as participants in Study 1 did. In other words, while there was an indication of improved cognitive empathy (accurately identifying the storyteller’s feelings), the social interest in the storyteller was not as affected by disclosing the autism diagnosis as it was in Study 1. As noted above, it could be that a larger sample size was needed to detect a smaller effect for Study 2’s participants. The results of Study 2 also support the conclusion that the effects cannot be explained solely by participants’ greater listening, their effort, or their attributed importance to performance, as these did not differ between conditions. Lastly, the results of Study 2 revealed a significantly enhanced willingness to work with the autistic person when knowing that he is autistic. The size of this effect (*d* = 0.85) was the largest of all results of both study 1 and study 2.

### Additional Analyses of Pooled Data from Studies 1 and 2

To assess the differences between STEM and non-STEM participants, we combined data from study 1 and 2 and conducted a series of two-way analyses of variance (ANOVAs) with introduction condition (Self- vs. No-disclosure) and STEM status (STEM vs. non-STEM) as independent variables. These analyses were performed for all shared dependent variables across both studies: empathic accuracy (both continuous valence and specific emotions), self-reported empathy, social interest (measured through interest in listening to an additional story and interest in meeting the storyteller), listening, effort, and attributed importance (measured through interest in knowing one’s accuracy). Prior to each analysis, outliers were identified and removed using a criterion of ± 2.5 standard deviations from the mean for each dependent variable. For significant interactions that emerged from the ANOVAs, we conducted follow-up analyses using estimated marginal means. Specifically, we performed planned contrasts to examine the effect of introduction condition separately within STEM and non-STEM groups. These contrasts were adjusted using Bonferroni correction for multiple comparisons to control for Type I error.

#### Results

*Empathic Accuracy - Specific emotions.* Analysis revealed a significant main effect of introduction condition (*F*_(1, 185)_ = 8.48, *p* =.004, *η²p* =.04). Participants in the self-disclosure condition demonstrated higher accuracy (*M* = 60.90, *SD* = 6.67) compared to those in the no-disclosure condition (*M* = 58.20, *SD* = 6.80). Neither the main effect of STEM status (*F*_(1, 185)_ = 0.31, *p* =.576, *η²p* =.002) nor the interaction between introduction condition and STEM status (*F*_(1, 185)_ = 0.09, *p* =.771, *η²p* <.001) were significant.

*Empathic Accuracy - Continuous valence*. Analysis revealed a significant main effect of introduction condition (*F*_(1, 184)_ = 4.37, *p* =.038, *η²p* =.02). Participants in the self-disclosure condition demonstrated higher accuracy (*M* = 0.69, *SD* = 0.22) compared to those in the no-disclosure condition (*M* = 0.62, *SD* = 0.26). Neither the main effect of STEM status (*F*_(1, 184)_ = 0.14, *p* =.710, *η²p* =.001) nor the interaction between introduction condition and STEM status *(F*_(1, 184)_ = 0.25, *p* =.615, *η²p* =.001) were significant.

*Self-Reported Empathy*. Analysis revealed a significant main effect of introduction condition (*F*_(1, 185)_ = 10.37, *p* =.002, *η²p* =.05), with participants in the self-disclosure condition reporting higher empathy (*M* = 6.57, *SD* = 1.63) compared to those in the no-disclosure condition (*M* = 5.75, *SD* = 1.88). There was a marginally significant main effect of STEM status (*F*_(1, 185)_ = 3.80, *p* =.053, *η²p* =.02), with non-STEM participants reporting somewhat higher empathy (*M* = 6.43, *SD* = 1.62) than STEM participants (*M* = 5.92, *SD* = 1.93). The interaction between introduction condition and STEM status was not significant (*F*_(1, 185)_ = 0.21, *p* =.644, *η²p* =.001).

*Social interest - Interest in listening to an additional story.* Analysis revealed a significant main effect of introduction condition (*F*_(1, 191)_ = 9.70, *p* =.002, *η²p* =.05), with participants in the self-disclosure condition reporting greater interest in listening to another story (*M* = 5.26, *SD* = 2.15) compared to those in the no-disclosure condition (*M* = 4.28, *SD* = 2.34). A significant main effect was also found for STEM status (*F*_(1, 191)_ = 6.77, *p* =.010, *η²p* =.03), with non-STEM participants reporting greater interest (*M* = 5.18, *SD* = 2.11) than STEM participants (*M* = 4.40, *SD* = 2.41). The interaction between introduction condition and STEM status was not significant (*F*_(1, 191)_ = 1.97, *p* =.162, *η²p* =.01).

*Social interest - Interest in meeting the storyteller*. Analysis revealed a significant main effect of introduction condition (*F*_(1, 191)_ = 6.63, *p* =.011, *η²p* =.05), with participants in the self-disclosure condition reporting greater interest in meeting the storyteller (*M* = 3.82, *SD* = 2.36) compared to those in the no-disclosure condition (*M* = 2.99, *SD* = 2.35). A significant main effect was also found for STEM status (*F*_(1, 191)_ = 15.64, *p* <.001, *η²p* =.03), with non-STEM participants reporting greater interest (*M* = 4.04, *SD* = 2.27) than STEM participants (*M* = 2.79, *SD* = 2.34). The analysis also revealed a marginally significant interaction between introduction condition and STEM status (*F*_(1, 191)_ = 2.80, *p* =.096, *η²p* =.01). Follow-up analyses using Bonferroni-corrected comparisons revealed that the effect of introduction condition was significant for non-STEM participants (*mean difference* = 1.41, *t*_(191)_ = 3.11, *p* =.004, *d = 0.45*), but not for STEM participants (*mean difference* = 0.33, *t*_(191)_ = 0.71, *p* =.957, *d = 0.10*).

*Listening*. Analysis revealed no significant main effects of introduction condition (*F*_(1, 188)_ = 0.87, *p* =.352, *η²p* =.005), or STEM status (*F*_(1, 188)_ = 0.21, *p* =.649, *η²p* =.001). The interaction between introduction condition and STEM status was also not significant (*F*_(1, 188)_ = 0.02, *p* =.876, *η²p* <.001).

*Effort.* Analysis revealed no significant main effects of introduction condition (*F*_(1, 186)_ = 1.61, *p* =.206, *η²p* =.009), or STEM status (*F*_(1, 186)_ = 1.98, *p* =.161, *η²p* =.01). The interaction between introduction condition and STEM status was also not significant (*F*_(1, 186)_ = 0.07, *p* =.790, *η²p* <.001).

*Attributed importance*. Analysis revealed no significant main effects of introduction condition (*F*_(1, 185)_ = 0.30, *p* =.582, *η²p* =.002), or STEM status (*F*_(1, 185)_ = 0.12, *p* =.728, *η²p* =.001). The interaction between introduction condition and STEM status was also not significant (*F*_(1, 185)_ = 0.39, *p* =.533, *η²p* =.002).

The results from the additional analysis of the agragated sample, as well as the discrepancy in the results of studies 1 and 2, highlights that the characteristics of the emphathizers play an important role in empathy towards, and social interest in, an autistic person and the impact of the disclosure of the diagnosis on those. We hypothesized that the results would be similar to those in Study 1 but attenuated, which was the case for the empathy measures but not for the measures of social interest. Similarly, the analysis of variance results suggests that while the disclosure of the diagnosis affected students from both groups’ empathy, in regard to social interest, it affected social sciences and humanities students but not the STEM students.

It could be, though, that not only the initial social vs. STEM orientation in interests affected these differences in results but also being a student, which involves being intensively focused on a field of study. In other words, it could be that while studying human behavior, one will be especially interested in meeting an autistic person, while students focusing on other subjects may not have this special interest. We, therefore, designed Study 3 to further explore the effect of the disclosure of autism on the empathy and social interest of non-autistic people outside the context of the university with non-student participants. While the question about willingness to work with an autistic person showed the strongest effect, the participants in study 2 were students and not engaged in a stable workplace. Study 3 was intended to replicate the results in the actual workplace.

## Study 3

In Study 3, we used data collected from a sample of participants in the workplace environment to examine the replicability of the findings outside of the university context with adults with diverse occupational backgrounds. We hypothesized that self-disclosure by an autistic storyteller will result in greater specific-emotions EA and greater self-reported empathy towards the autistic storyteller. Regarding social interest, we did not have an apriori hypothesis. Since this sample included actual working adults, the question about willingness to work with the target was especially important.

### Methods

#### Participants

Participants were recruited through postings in a newsletter of a major high-tech company and invitations distributed to workers in any steady workplace through social media and word of mouth. The final sample included both STEM-oriented professions (48.68%), social-oriented (psychologists, social workers, etc., 38.16%), and others (e.g., artists; 13.16%). Eighty-one participants completed the study procedure, and 5 of them were excluded (one participant reported a mental health condition without detailing, 3 reported not being native speakers of the language used, and one participant reported technical problems during participation). Final analyses were conducted on 76 non-autistic participants (50% females, 50% males, *M*_age_=40.65, *SD*_age_=14.22).

#### Procedure and Design

The procedure and design were similar to that in Studies 1 and 2, such that participants were randomly assigned to one of the two conditions: (1) *Self-Disclosure* (*n* = 40; 47.5% females) or (2) *No-Disclosure* (*n* = 36; 50% females). Measures and data analysis were identical to those used in Study 2.

### Results and Discussion

The results revealed significant differences between the conditions for the specific emotions’ EA measure (*t*_(74)_ = 3.55, *p* = .001, [*adjusted p* = .002], *d* = 0.81), self-reported empathy (*t*_(58)_ = 2.26, *p* = .028 [*adjusted p* = .039], *d* = 0.53), and an interest in listening to an additional story (*t*_(71)_ = 3.41, *p* = .001 [*adjusted p* = .007], *d* = 0.78). A marginal difference between the conditions was found for the continuous valence EA measure (*t*_(73)_ = 1.80, *p* = .076, *d* = 0.29) and also for interest in meeting the storyteller (*t*_(74)_ = 1.66, *p* = .068, *d* = 0.42). These findings indicate higher specific-emotions EA and self-reported empathy in the self-disclosure condition compared to the no-disclosure condition, more interest in hearing more from the storyteller, and a similar trend for accurately tracking the valence of his feelings while storytelling and interest in meeting him in real life, with a marginal result that did not reach significance in these measures. Consistent with Study 2’s findings, the results of Study 3 also indicated more willingness to work with the storyteller in the same (hypothetical) work team on the part of those who heard the storyteller disclosing his autism compared to those who did not (*t*_(74)_ = 3.07, *p* = .003 [*adjusted p* = .010], *d* = 0.70). Unlike in Studies 1 and 2, the results of Study 3 also demonstrated a higher level of self-reported listening to the story (*t*_(51)_ = 2.51, *p* = .015 [*adjusted p* = .036], *d* = 0.58;) and an effort to infer the storytellers’ emotions (*t*_(71)_ = 2.38, *p* = .020 [*adjusted p* = .035], *d* = 0.55) on the part of participants in the self-disclosure condition. Similar to Studies 1 and 2, no difference between the conditions was found in the participants’ attributed importance to performance (Table 3 presents the full analysis).

**Table 3:** Comparisons between the Self-Disclosure and the No-Disclosure conditions for Empathic Accuracy and Self-Reports measures in study 3

	Parameter	Self-Disclosure (*n*)	No- Disclosure (*n*)	Mean Self-Disclosure	Mean No- Disclosure	df	t	*p*	Adjusted *p*	d
**Empathic Accuracy**	Continuous valence	40	36	0.66	0.57	73	1.80	*0.076*	*0.076*	0.29
	Specific emotions	40	36	61.69	55.99	74	3.55	**0.001**	**0.002**	0.81
**Self-Reports**	**Empathy**	40	35	6.77	5.91	58	2.26	**0.028**	**0.039**	0.53
	**Social interest**: Interest in listening to an additional story	40	36	5.4	3.58	74	3.42	**0.001**	**0.007**	0.78
	**Social interest**: Interest in meeting the storyteller	40	36	3.82	2.77	74	1.66	*0.068*	0.080	0.42
	**Social interest**: Interest in working with the storyteller in a workplace	40	36	4.95	3.41	74	3.07	**0.003**	**0.010**	0.70
	**Listening**	40	36	7.45	6.75	51	2.51	**0.015**	**0.036**	0.58
	**Effort**	39	34	7.15	6.41	71	2.38	**0.020**	**0.035**	0.55
	**Attributed importance**: Interest in knowing how accurate you were	38	35	6.81	6.40	71	1.22	0.226	0.226	0.28

*Adjusted p– p values after Bonferroni correction, with separate adjustments for the behavioral Empathic Accuracy scores and self-report measure

#### General Discussion

The three studies presented focused on non-autistic people’s empathy and social interest towards an autistic person and the role of the disclosure of the diagnosis in these processes. The results of the first study, conducted with students in the humanities/social sciences, indicated a positive effect of autism self-disclosure on accurately rating the emotions of an autistic person according to the way he himself rated them (empathic accuracy). The results of the second study conducted with students in STEM implied a similar trend but with marginal significance and smaller effect sizes. In both studies, there was also an indication for greater self-reported empathy (marginal significance in Study 2). Results of Study 2 indicated another marginal effect on an additional measure of empathy: the participants’ performance in rating the valence of the autistic person’s positive to negative feelings in concordance with the storyteller’s own ratings (continues empathic accuracy). The additional analysis combining both student samples (Studies 1 and 2) provided further insights into these patterns. For empathy, we found that the disclosure of the autism diagnosis impacted three measures: specific emotions empathic accuracy, continuous valence empathic accuracy, and self-reported empathy. There were no significant interactions between disclosure and field of study, indicating that knowing the storyteller was autistic improved empathy similarly for both STEM and non-STEM students, even though the magnitude of this improvement varied.

The pattern for social interest measures revealed a more complex picture. While the disclosure showed a significant positive effect overall, there was also a difference between student groups: non-STEM students showed greater interest than STEM students across conditions, perhaps reflecting their general orientation toward social and interpersonal topics. Moreover, the effect of disclosure on social interest was particularly strong for non-STEM students, suggesting they were especially motivated to connect with the storyteller when they knew he was autistic. These results indicate that while disclosure consistently enhanced empathy regardless of field of study, its effect on social interest was influenced by students’ academic background and potentially their pre-existing interests in social interaction.

The results of Study 3, which involved older, non-student participants already immersed in a workplace, also indicated enhanced empathy towards an autistic person when disclosing his autism compared to when not. Together, these results imply a positive effect of the disclosure of an autism diagnosis by an autistic person on non-autistic observers’ empathy towards them. As some of these effects did not hold after controlling for multiple comparisons, findings should be interpreted cautiously, and replications based on larger samples are needed.

One explanation for the positive impact of self-disclosure on empathy towards the autistic discloser could be increased attention. It could be that when people are presented with the disclosure, they are more attentive, thus performing better in inferring the storyteller’s feelings. The results of Study 3 indeed indicate greater reported listening and effort on the part of the participants in tracking the storyteller’s emotions when knowing he was autistic. However, in Studies 1 and 2, participants who did or did not know that the storyteller was autistic did not differ in self-reporting their listening or effort to empathize, and in all three studies, attributed importance to their performance in empathizing with the storyteller did not differ whether they knew the person was autistic or did not know. Thus, self-reports of the participants’ own experience do not fully support enhanced attention as an explanation for the effect found. Nonetheless, it is possible that enhanced attention occurred, but participants are not always aware of it. Future studies should directly examine this hypothesis, for example, by adding an attention task, questions, or physiological measures.

While social desirability could explain some of our findings, several patterns in our data suggest it cannot fully account for the effects. If results were purely due to social desirability, we would expect to see effects mainly in self-report measures. However, we found improved performance in the behavioral empathic accuracy measure, which would be more difficult to consciously manipulate. Additionally, if social desirability was the main driver, we would expect to see enhanced scores across all self-report measures when autism was disclosed. However, some measures (like attributed importance to performance) showed no differences between conditions across studies. Nevertheless, social desirability likely played some role in our findings, particularly in self-reported measures of social interest, and this may have been especially true for participants studying human behavior (Study 1), who might have felt more pressure to appear accepting of neurodiversity. Future studies using implicit measures could help further disentangle genuine effects from social desirability influences.

Another possible explanation for the higher empathy when knowing that the storyteller was autistic might be an overall effect of disclosing personal information - the disclosure of personal information was found to increase liking of the other for both the discloser and the disclosed (Sprecher et al., 2013). This hypothesis, again, calls for a future direct examination. It is worth mentioning, though, that the stimulus itself was an autobiographical story, disclosing personal information by the storyteller, and the conditions differed only in the additional statement of autism disclosure. In future studies, it would be interesting to compare the disclosure of autism to that of another piece of information or to use another task.

A fourth, and perhaps the most compelling explanation for the positive effect of autism self-disclosure on empathy towards the discloser might be that knowing that the storyteller is autistic allows the listener to look at them differently. Previous studies have shown that non-autistic individuals struggle with understanding autistic individuals’ facial expressions and body movements (Brewer et al., 2016; Edey et al., 2016) and may interpret them as being awkward. However, knowing in advance that the storyteller is autistic might allow listeners to attribute “awkward” expressions to the autism and be more focused on the content and the storyteller’s emotions rather than trying to understand different patterns of communication. Furthermore, previous studies indicated that non-autistic observers’ first impressions of autistic individuals engaging in real-world social behavior were less favorable than those of non-autistic controls (Sasson et al., 2017) and that first impressions of autistic adults improve with diagnostic disclosure (Sasson & Morrison, 2019). It could be that knowing that a person is autistic mediates or moderates the effect of less favorability of autistic people and that non-autistic observers are less negatively affected implicitly when the target’s autism is disclosed. This means that perhaps the disclosure does not increase empathy towards the autistic discloser but decreases the ‘less favorability effect,’ which interferes with empathizing with an autistic person when their autism is not disclosed.

Interestingly, while the results of Study 2 revealed a similar trend to those of Studies 1 and 3 regarding a positive effect of autism disclosure on empathy, they did not for social interest. Participants in Study 2 did not differ in their reports of how interested they were in hearing another story from the autistic storyteller or in meeting him, depending on whether they did or did not know that the storyteller was autistic. Participants in Study 1 were students studying psychology, education, social work, and other subjects classified under the broad social sciences or humanities categories. It could be that these individuals who chose to study human behaviour were more interested in learning about an autistic person’s experiences than individuals who chose to study STEM. This fits with previous work showing that students of humanities and social sciences score higher on measures of empathy compared to students in STEM (Wakabayashi et al., 2006; Wheelwright et al., 2006). It could also be that Study 1’s participants were more prone to social desirability, i.e., they wanted to appear more interested when knowing that the storyteller is autistic, compared with participants in Study 2, or felt that being interested in an autistic person is expected from them based on their studies.

However, the greater social interest in the autistic person was also evident in Study 3, which included a heterogeneous sample from the general population. Importantly, while STEM students were not affected by the self-disclosure statement in expressing social interest in the autistic person, results did not go in the other direction. This points to another possible explanation: It could be that the STEM students attributed less importance to the fact that a person was autistic, i.e., that “was not an issue” for them when it comes to social interest– as knowing this fact did not improve or reduce their social interest in him. In future studies, it will be interesting to examine if STEM students are exposed more than others to autistic people as colleagues or family members or have higher autistic traits themselves and if that is associated with being less affected by knowing that a person is autistic in showing interest in them. The differences between the results of the three studies indicate that the effect of self-disclosure on social interest towards the autistic discloser is complex and depends on the characteristics of the person being disclosed to. Importantly, there was no measure for which self-disclosure resulted in a negative outcome on social interest in the autistic disclosure across the three samples.

Both STEM students in Study 2 and working adults in Study 3 were affected by the disclosure of autism when reporting their willingness to work with the storyteller. This could be explained by the fact that expressing a willingness to work with someone also involves moral and ethical considerations, such as not wanting to demonstrate discrimination towards an autistic person. This echoes previous findings about more favorable ratings of interviewees in job interviews when autism is disclosed (Norris et al., 2024). Further research should investigate whether this effect replicates in other implicit behavioral measures that do not rely on self-report and if these “non-binding” reports regarding hypothetical situations will translate into actual, “real life” decision-making.

While our findings show improved empathy in response to a single autobiographical story, the translation of this immediate empathic response into lasting behavioral change remains an open question. The gap between hypothetical willingness to work together and actual workplace behavior, or between momentary empathic accuracy and sustained mutual understanding, warrants further investigation. Future research should examine whether these initial empathic responses persist over time and generalize to different contexts and interactions.

The studies reported here have several limitations. First, although the stimulus was based on the lived experience of an autistic person and his natural facial expressions, watching a pre-recorded video may not fully reflect the effect of self-disclosure during live interactions. Future research using live interaction designs would be beneficial in gaining a better understanding of the questions examined. Second, we used a stimulus based on one (verbally and intellectually capable) autistic person. Although we were interested in the effect of self-disclosure on (three samples of) non-autistic participants, further replications based on additional individuals or other stimuli (for example other contents of autobiographical stories) are essential for evaluating the results’ robustness and generalisability. However, at the same time, the use of singular stimuli strengthens the conclusions on the differences between the samples of empathizers (which the current study puts in the center of focus), as they were all exposed to the exact same stimuli. Future studies should incorporate various disclosures/targets of empathy and populations of observers/empathizes in the same design, allowing greater generalizability and an in-depth investigation of the possible interplay between the disclosures’ and the empathizers’ characteristics. This would, of course, require much larger sample sizes. In addition, while our main focus was on empathic accuracy (and self-reported empathy, as a whole), other facets of empathy should be measured in future studies, for example, empathic care and concern.

In future studies, it will also be interesting to have independent raters assess the degree to which storytellers in the stimulus videos exhibit autistic traits, as participants’ perceptions may be affected depending on the degree to which Gricean maxims are violated. It also should be noted that participants across the three studies were either university students or professionals, and it could be that these populations are more accepting of differences than other populations. Importantly, analysis considering whether participants had any previous knowledge about or experience living with autistic people would also be of great value.

Additionally, sample sizes of females and males differed between Studies 1 and 2, and future studies should examine whether patterns differ by sex/gender. The sex ratio was even in Study 3, but the sample size was not large enough to allow the examination of the effects of sex, condition, and the interaction between them, a question worthy of further examination. As the storyteller described a past romantic experience, participants’ responses about meeting the storyteller might have been influenced by their own romantic interest or their interpretation of whether such interest was relevant to the question. However, given that when an impact of disclosure was observed across measures of empathy and social interest, it showed consistent directionality, this potential confound seems less likely - or if it did have an impact, it worked in the same direction as other effects. Future studies should include questions about participants’ sexual orientation and could examine whether self-disclosure affects romantic interest specifically.

Lastly, we relied on participants’ self-reports of being non-autistic. Although it is likely to be uncommon, some participants may have chosen not to disclose their autism or may have been unaware that they are autistic. Future studies could examine these questions by directly measuring autistic traits. Importantly, it will also be interesting to examine how self-disclosure of an autism diagnosis impacts autistic people. Notwithstanding these limitations, the current project provides evidence across three studies that self-disclosure of autism positively affects non-autistic people’s empathy and flags the role of the empathiser’s own characteristics in the complex effect of self-disclosure on social interest towards an autistic person.

The fact that disclosure of the autism diagnosis improved non-autistic participants’ empathic accuracy suggests that some of the mutual misunderstanding described by the double empathy problem theory can be mitigated by awareness of neurotype differences. This raises intriguing questions about the nature of the empathy gap between autistic and non-autistic individuals. If knowledge of neurotype differences can enhance understanding, this suggests that the communication breakdown might be partially addressed through increased awareness and explicit acknowledgment of different communication styles, rather than being solely due to inherent neurological differences. These findings might also have implications for understanding the experiences of late-diagnosed autistic adults; for example, receiving a diagnosis could not only help them understand their own experiences but also facilitate others’ understanding of their emotional expressions. This interpretation, which suggests potential pathways for bridging the empathy gap, should be further explored in future research.

Importantly, someone’s decision to disclose their autism should not solely depend on the effect it might have on the other’s empathy and social interest toward them (Farsinejad et al., 2022; Grandin, 2004; Romualdez et al., 2021; Thompson-Hodgetts et al., 2020). The process of self-disclosing personal information can be gratifying, as it has been found to activate the reward regions of the brain (Tamir & Mitchell, 2012). Self-disclosure might positively impact an autistic person through empowerment or relief, and it was previously found that the effort of camouflaging autistic behaviours has negative effects on the well-being of autistic people (Cook et al., 2021). On the other hand, self-disclosure could also negatively affect the discloser, for example, by causing anxiety. Indeed, some autistic people advocate for self-disclosure, while others fear it will lead to stigma and rejection (Farsinejad et al., 2022; Romualdez et al., 2021; Thompson-Hodgetts et al., 2020). The individual’s decision of to whom, when, how, and what to disclose might vary for the same person in different contexts (Chaudoir & Fisher, 2010; Greene et al., 2006). Nevertheless, considering the “double empathy problem” framework, the positive impact that autism disclosure is likely to have, specifically on non-autistic people’s *empathy* towards an autistic person, should be taken into account when considering the disclosure of an autism diagnosis in social interactions.
